# Upgrading COCATS: A Fellow’s Perspective on Practical AI Integration With Cardiovascular Fellowship Training

**DOI:** 10.1016/j.jacadv.2024.101367

**Published:** 2024-10-25

**Authors:** Najah Ali Khan

**Affiliations:** Scripps Memorial Hospital, La Jolla, California, USA

**Keywords:** artificial intelligence, clinical practice, fellow, medical education, research

Numerous studies underscore artificial intelligence (AI)'s growing role in diagnostics and predictive analytics, leveraging tools like electrocardiogram, echocardiograms, cardiac computed tomography, genetic studies, and biomarkers.^1^, ^2^, ^3^, ^4^ Recent advancements, including machine learning models that incorporate clinical data, genomics, and wearable sensor data, are emerging as powerful tools for predicting individual patient risk and optimizing treatment strategies.^1^^,^^3^, ^4^, ^5^, ^6^, ^7^, ^8^, ^9^, ^10^ However, integrating AI into clinical practice comes with significant challenges, including concerns about data privacy, model interpretability, and real-world performance, especially in settings different from those where the algorithms were initially developed.^2^^,^^3^ Ensuring responsible use in cardiology requires rigorous validation and transparent communication about the capabilities and limitations of these AI tools.

How can we tackle these challenges? From my perspective as a fellow, it is essential to embrace AI's transformative potential in cardiology, particularly given the rapid pace of knowledge advancement in the field. This requires a strong educational and experiential foundation in AI for cardiovascular trainees and early career investigators. They must be equipped to use AI for formulating research questions, understanding key principles of cardiovascular medicine, and integrating AI into patient care. Collaboration with AI experts and data scientists is also vital to drive innovation within training programs.

I encountered significant time and energy constraints when starting cardiology fellowship. Moreover, many colleagues had little to no formal exposure to AI and machine learning. This lack of familiarity led to hesitation in adopting digital health tools, largely due to uncertainty about their utility, a lack of understanding of the development process, and fears of replacing human-centered care. Additionally, I found the tools I learned from my graduate studies in biomedical informatics were quickly becoming outdated, necessitating continuous learning, especially in evolving areas like prompt engineering. Recognizing these challenges, I sought practical AI applications for AI in clinical and research settings, applying these tools in real time. Initially, finding new AI tools was difficult, but I began exploring unconventional sources. I followed leading researchers on platforms like X (formerly Twitter), Instagram, and Reddit, which exposed me to various AI tools and their applications (Table 1).

**Table 1:** AI Tools Utilized in Clinical Practice, Medical Education, and Research Training: Application Uses and Learning Outcomes

Category	AI tool	How I found it	Application use	Learning outcome
Clinical practice	AI scribe software (Scribe Note, ChartNote)	Reddit	Automates SOAP notes from patient conversations.	Reduced documentation time, improved patient communication.
Clinical practice	ChatGPT (ER Differential)	Personal experimentation and recommendations from colleagues	Aids in considering differential diagnoses outside specialty.	Enhanced diagnostic accuracy in emergency care.
Clinical practice	ChatGPT 4o (Real-time Translation)	X	Enables real-time communication with non-English speaking patients.	Improved care delivery while awaiting a translator.
Clinical practice	ChatGPT 4 (Difficult Conversations)	Reddit	Offers guidance on handling complex patient conversations.	Enhanced patient understanding and decision-making.
Clinical practice	ChatGPT 4 (Hemodynamic Calculations)	Personal experimentation	Assists with quick, accurate hemodynamic calculations.	Increased efficiency in patient management.
Clinical practice	ChatGPT 4 (Object Recognition)	Instagram	Identifies medical tools and conditions from images.	Enhanced diagnostic accuracy and preparedness.
Medical education	ChatGPT 4o (Flashcards)	Instagram	Generates study materials like flashcards.	Efficient board exam preparation.
Medical education	ChatGPT 4o and Claude 3 (Study Plans)	Instagram	Helps design personalized study plans.	Improved educational outcomes for learners.
Medical education	ChatGPT 4 (Procedure Walkthroughs)	Personal experimentation	Guides procedural steps and interpretations.	Better understanding and teaching of procedures.
Research training	Jenni AI	Instagram	Assists with writer's block and finding references.	Enhanced research productivity.
Research training	ChatGPT-4, 4o, and Claude 3 (Julius AI)	Identified through research on AI tools for data analysis	Enables comprehensive data analysis and visualization.	Streamlined research processes and better outputs.
Research training	ChatGPT 4 (Email Drafting)	Colleagues‘Äô recommendations and personal exploration	Assists in drafting clear and concise emails.	Improved communication and project management.
Research training	Otter.AI	Reddit	Provides live transcription and meeting summaries.	Better documentation and project organization.
Research training	Reclaim AI	Google	Automates time blocking and project scheduling.	Optimized time management in a busy schedule.
Research training	Notion AI	X	Organizes research projects and aids in protocol development.	More efficient IRB submissions and project management.
Research training	Consensus AI, SciSpace, Scite.AI, OpenEvidence	X	Streamlines literature reviews and study summaries.	Improved efficiency and depth in literature reviews.
Research training	ChatGPT-4 and Beautiful.AI (Presentations)	Google	Enhances presentation creation and design.	High-quality, efficient presentations.

AI = artificial intelligence; IRB = institutional review board; SOAP = Subjective, Objective, Assessment, and Plan.

I've leveraged AI in patient communication, particularly in complex or urgent scenarios. In a case involving a patient with cardiac tamponade who spoke only Arabic, I used ChatGPT 4o to facilitate a real-time, bidirectional conversation while my team arranged for a human translator. This ensured the patient received timely and accurate information. When discussing limited treatment options with an amnestic patient post-myocardial infarction, ChatGPT 4o provided effective communication strategies and specific phrasing to convey complex information in a compassionate and understandable manner. This enhanced patient understanding and improved the overall quality of care.

During my busy heart failure rotation, time is often limited, yet precise hemodynamic calculations are essential for accurate patient assessment and management. By using ChatGPT 4o, I can quickly input nonpatient identifying right heart catheterization measurements and receive accurate calculations for parameters such as Transpulmonary Gradient, Diastolic Pulmonary Gradient, Pulmonary Artery Pulsatility Index, Fick Cardiac Output/Index, and Cardiac Power Output. Reviewing the step-by-step calculations by the AI, I can verify the results, saving time and ensuring accuracy. This integration of AI into routine clinical practice significantly enhanced my efficiency and confidence in making data-driven decisions with good patient outcomes.

One of the most productive applications of AI in my research has been in the field of data analytics. I worked on a project involving unstructured data from text impressions of imaging studies. I made a workflow of how I was going to clean and analyze the data. I downloaded a subset of the text impressions along with their corresponding anonymized IDs into an Excel sheet. After relabeling the headers and formatting the data to make it more accessible for AI interpretation—by removing all formatting characters—I uploaded the refined data set into Julius AI, a data-analytics platform that uses advanced models like ChatGPT-4o and Claude 3. Using a predefined workflow for data cleaning, Julius AI suggested steps such as tokenization, stop word removal, and lemmatization, which are standard practices in natural language processing. Once the data were cleaned, I instructed Julius AI to extract specific measurements and classifications, organizing them into separate columns. I then asked Julius AI to validate its findings by reviewing the underlying code to ensure accuracy.

Julius AI proved equally effective with structured numerical data, such as the Integrated Public Use Microdata Series National Health Interview Survey data set. I started by downloading the data set in Excel, relabeling the headers, and reformatting the data to improve AI interpretation—converting numerical three-digit codes into binary or single-digit variables. After uploading the refined Excel document to Julius AI, I used a workflow to clean the data. Julius AI identified cells with missing data, suggested ways to clarify the data set, and filled in missing values where appropriate. It then generated descriptive statistics, bar graphs, and performed statistical tests such as Fisher’s exact test, culminating in a forest plot of the results. Again, Julius AI provided the exact Python code used in the analysis, which saved me considerable time in data analysis and visualization while ensuring transparency in the methodology.

I use ChatGPT-4 and Claude 3 to develop medical education materials. I uploaded study documents for my general cardiology board question bank and asked the AI models to generate flashcards as a character separated values file based on the content. This resulted in a comprehensive Excel file I imported onto a flashcard application for board exam preparation. Additionally, I use AI tools to design study plans and conceptual frameworks for medical students and residents on service. ChatGPT-4 helps me create tailored teaching outlines for specific topics on rounds, producing study materials residents and students can reference later. I took a picture of a patient monitor (without patient health information), and the AI models recognized the right heart catheterization waveform measurements. I then asked it to create a step-by-step interpretation guide that my resident could use (after reviewing its content, of course). I also used it to understand the steps involved in performing and interpreting right heart catheterization results, clarifying steps I was unfamiliar with my attending.

Integrating AI into education and clinical practice highlights a significant gap in current cardiology education frameworks, such as the COCATS (Core Cardiovascular Training Statement). COCATS was last updated in 2015. Since then, AI has made significant improvements in medical knowledge, patient care, systems-based practice, practice-based learning and improvement, professionalism, and interpersonal and communication skills, which are core COCATS competencies. Based on my experiences in AI, I believe COCATS should incorporate the following: 1) foundation in AI, machine learning, and model development; 2) clinical applications of AI; 3) regulatory and ethical considerations; 4) human-AI collaboration and clinical workflow optimization; and 5) AI in research, education, and continuous learning.

Integrating AI into cardiology training involves a comprehensive approach, starting with a strong foundation in AI, machine learning, and model development. Fellows should begin by learning the differences between supervised learning (eg, using labeled data sets to train an AI model to predict heart failure based on patient history) and unsupervised learning (eg, clustering patients into risk groups based on their cardiovascular profiles without predefined labels). They should explore key algorithms like neural networks, which are used in deep learning models for interpreting complex cardiac imaging data, and understand issues like overfitting, such as when a model predicts heart disease well in the training data but fails in new, unseen data. They must also become proficient in performance metrics, such as understanding how the area under the receiver operator characteristic curve helps determine a model’s ability to distinguish between patients with and without atherosclerosis.

As fellows advance, they should delve into clinical applications of AI, exploring how AI enhances cardiovascular care. They might study AI- tools that analyze echocardiograms to automatically measure left ventricular ejection fraction, improving diagnostic accuracy and efficiency, which is already well studied.^1^ They could also learn how AI integrates with electronic health records to provide real-time alerts for potential adverse cardiac events based on patient data trends.^3^ In personalized medicine, fellows might examine how AI algorithms analyze genetic data from big data sets, like all of us, to predict individual responses to medications like beta-blockers, allowing for more tailored treatment plans.^5^

Understanding regulatory and ethical considerations is important for the responsible use of AI in health care. Fellows should become familiar with the Food and Drug Administration’s guidelines for approving AI-based medical devices, such as the requirements for showing an AI tool accurately predicts coronary artery disease across diverse populations.^6^ They should also explore ethical scenarios, such as ensuring AI algorithms used for predicting stroke risk do not inadvertently introduce bias against certain patient groups by considering factors like race or socioeconomic status without proper context.^2^

Fellows should further focus on human-AI collaboration and clinical workflow optimization. They might participate in projects where AI helps clinicians by automating routine tasks like sorting through large volumes of cardiac magnetic resonance imaging data, letting doctors focus on more complex decision-making.^7^ They’ll learn to work alongside data scientists to refine these tools, making sure AI recommendations are accurate and actionable in a clinical setting. For example, this collaboration may lead to AI triaging outpatient messages based on patient risk profiles, optimizing the workflow in busy cardiology departments without disrupting patient care but ensuring timely care.^8^

AI in research, education, and continuous learning will keep fellows at the cutting edge of cardiology. For example, they might use AI to analyze large data sets in a research study, identifying novel predictors of heart failure outcomes.^9^ In education, fellows could engage with adaptive learning platforms that use AI to customize study materials based on their strengths and weaknesses, ensuring a more efficient learning process.^10^ Continuous engagement with AI, such as attending workshops on the latest advancements in AI-driven cardiac care, will ensure they remain informed and capable of leveraging new technologies.^4^

Integrating AI into cardiology offers transformative potential, enhancing clinical practice, medical education, and research, but must be done with careful ethical, technical, and regulatory considerations. For AI to realize its promise, comprehensive education and practical training in AI concepts and applications are essential for cardiovascular trainees and early career investigators. As we look to the future, the ongoing integration of AI into medical training and practice will shape the evolution of cardiology. By embracing AI critically and informedly, we can harness its potential to enhance patient outcomes, advance research, and elevate the standards of cardiovascular care.

## Funding support and author disclosures

The author has reported that they have no relationships relevant to the contents of this paper to disclose.
